# Combining AZD8931, a novel EGFR/HER2/HER3 signalling inhibitor, with AZD5363 limits AKT inhibitor induced feedback and enhances antitumour efficacy in HER2-amplified breast cancer models

**DOI:** 10.3892/ijo.2015.3062

**Published:** 2015-06-22

**Authors:** CLAIRE CRAFTER, JOHN P. VINCENT, ERIC TANG, PHILLIPPA DUDLEY, NEIL H. JAMES, TERESA KLINOWSKA, BARRY R. DAVIES

**Affiliations:** 1 Oncology iMED, AstraZeneca, Alderley Park, Macclesfield SK10 4TG, UK; 2 Oncology iMED, AstraZeneca, CRUK Cambridge Institute, Cambridge CB2 0RE, UK

**Keywords:** AZD5363, HER2-amplified breast cancer, PI3K/AKT/mTOR, combination therapy, feedback

## Abstract

The phosphatidylinositol 3-kinase (PI3K)/AKT/mammalian target of rapamycin (mTOR) signalling network is frequently de-regulated in breast cancer and has been shown to mediate resistance to anti-HER2 agents. Whilst constitutive activation of this pathway is emerging as a marker of sensitivity to various PI3K pathway inhibitors, activity of these agents in the clinic may be limited by the presence of feedback loops, leading to reactivation of receptor tyrosine kinases, such as HER2/HER3. To determine whether inhibition of HER2 could increase the efficacy of AZD5363, a novel AKT inhibitor, a panel of breast cancer cells was dosed with AZD5363 in combination with AZD8931, an inhibitor of EGFR/HER2/HER3 signalling. We show that the combined treatment resulted in synergistic growth inhibition and enhanced cell death, specifically in the HER2-amplified cell lines. Investigation of the mechanism by western blot analysis revealed that the addition of AZD8931 prevented the induction of HER2/HER3 phosphorylation induced by AZD5363 and resulted in concomitant inhibition of both the PI3K/AKT/mTOR and ERK signalling pathways and induction of apoptosis. Using the HCC1954 xenograft model, which is resistant to trastuzumab, we show that the combination of AZD5363 and AZD8931 is more efficacious than either agent alone, resulting in profound tumour regressions. We conclude that the activity of AZD5363 in HER2-amplified breast cancer cells is enhanced by the addition of AZD8931 and that dual targeting of AKT and EGFR/HER2/HER3 signalling is an attractive treatment option to be explored in the clinic.

## Introduction

The phosphatidylinositol 3-kinase (PI3K)/AKT/mammalian target of rapamycin (mTOR) axis lies downstream of receptor tyrosine kinases (RTKs) and plays a key role in regulating critical cellular processes such as growth, metabolism, size, motility and survival (1,2). This signalling network is reported to be the most commonly altered pathway in human cancers and activation can occur through several different mechanisms. In breast cancer these include, overexpression/amplification of HER2 (ErbB2/neu), activating mutations in PIK3CA, the gene encoding the α catalytic subunit of PI3K, mutation or amplification of AKT and loss of the lipid phosphatase, phosphatase and tensin homolog (PTEN) (3). Several preclinical studies have suggested that activation of the pathway, either through PIK3CA mutations or PTEN loss, can predict sensitivity to a range of PI3K pathway inhibitors, targeting different nodes on the signalling axis (4–7). Data are also emerging from the clinic to suggest that patients with PIK3CA mutations, treated with PI3K/AKT/mTOR inhibitors, demonstrate a higher response rate than patients without mutations (8,9).

Despite the initial activity of PI3K pathway inhibitors, resistance is likely to occur, as has been seen with other targeted therapies in the clinic (10,11). Normal activation of PI3K signalling is regulated by feedback inhibition of upstream components of the pathway such as IRS1 and RTKs (reviewed in ref. 12). It is becoming increasingly evident that PI3K pathway inhibitors relieve this feedback, which may therefore limit their anti-tumour activity. For example, treatment with rapamycin, the mTORC1 inhibitor, inhibits the phosphorylation and activation of p70S6K and disrupts the negative feedback loop to IRS-1 and Grb10 (13–15). This leads to activation of insulin-like growth factor-1 receptor (IGF-1R) and re-phosphorylation and activation of AKT. Furthermore, additional feedback mechanisms, involving FOXO transcription factors, have subsequently been identified in HER2-amplified and ER^+^ breast cancer cells (16–18). Inhibition of AKT results in the activation of FOXO-dependent gene transcription of multiple RTKs, including IGF-1R and HER2/HER3, leading to reactivation of PI3K signalling as well as activation of compensatory pathways such as ERK1/ERK2. These data suggest that targeting the PI3K pathway in combination with anti-HER2 agents would be superior to monotherapy treatment. This is further supported by the fact that hyperactivation of the PI3K pathway, either through mutations in PIK3Ca or loss of PTEN, has been associated with resistance to HER2-targeted therapies such as trastuzumab and lapatanib (19–22). Consistent with these observations, we and others have demonstrated enhanced anti-tumour efficacy in preclinical models of HER2-amplified breast cancer by combining PI3K pathway inhibitors with trastuzumab or lapatinib (7,16,23–26).

In this study, we sought to extend these findings, to demonstrate the mechanism of enhanced activity in combination and also to determine whether a similar effect could be observed with the novel inhibitor AZD8931, which displays equipotent activity against EGFR, ErbB2 (HER2) and ErbB3 (HER3) signalling (27). We show that AZD5363 in combination with AZD8931 causes profound shutdown of the PI3K signalling pathway as well as concomitant inhibition of the ERK pathway. This resulted in decreased proliferation and enhanced cell death in a range of HER2-amplified breast cancer lines *in vitro* and tumour regression *in vivo*. These data indicate that combining AZD5363 with a HER2 agent represents an attractive therapeutic strategy in the clinic for the treatment of HER2-amplified breast cancer.

## Materials and methods

### Cell lines and treatments

All cell lines were maintained at 37°C and 5% CO_2_ in a humidified atmosphere and grown in RPMI-1640 growth media supplemented with 10% FBS and 2 mmol/l glutamine. The BT474c cell line was subcloned from the human breast cell line BT474, provided by Drs J. Albanell and J. Baselga, Laboratorio Ricerca Oncologica, Barcelona, Spain. BT20 cells were purchased from the European Collection of Cell Cultures, SUM52PE were from Asterand and the remaining lines were all from the American Type Culture Collection. The identity of all cell lines was confirmed using short tandem repeat analysis as previously described (7). AZD5363, AZD8931 and AZD6244 were dissolved in DMSO to a concentration of 10 mmol/l and stored under nitrogen.

### Determination of cell growth

Cells were seeded in 384-well black, clear bottomed plates (Greiner Bio-One, Stonehouse, UK) at 1,000–2,000 cells per well, cultured for 18–24 h and treated with increasing concentrations of AZD5363 and AZD8931 (0.03–3 μmol/l) in a 6×6 dosing matrix. After 5 days of treatment, live cell number was determined using a Sytox Green endpoint as previously described (7). Briefly, Sytox Green nucleic acid dye (Invitrogen) diluted in TBS-EDTA buffer was added to cells at a final concentration of 0.13 μmol/l and the number of dead cells detected using an Accumen Explorer (TTP Labtech, Melbourn, UK). Cells were then permeabilised by the overnight addition of saponin (0.03% final concentration, diluted in TBS-EDTA buffer) and a total cell count measured. The live cell count was then determined by subtracting the number of dead cells per well from the total number of cells. Pre-dose measurements were made to indicate the number of live cells at the start of the experiment and thus an indication of whether the treatment regimen had resulted in cell death. The data are presented as % growth using the NCI formulas as follows: [(Ti-Tz)/(C-Tz)] ×100 for values for which Ti≥Tz and [(Ti-Tz)/Tz] ×100 for concentrations for which Ti<Tz where, Tz represents the number of live cells at time zero, C represents the control growth and Ti represents the number of live cells in the presence of each drug regimen. This formula gives a growth percentage from −100% to +100%. Negative scores are for cell killing and positive scores are for anti-proliferation. Experiments were performed in triplicate.

### Analysis of combination activity

Two dimensional dose response matrix and curve fitting were processed in the combination extension of Genedata Screener12™ (Genedata, Basel, Switzerland). To enable this, % growth values were converted to a positive scale using a modified NCI formula as follows: [1-(Ti-Tz)/(C-Tz)] ×100 for values for which Ti≥Tz and [1-(Ti-Tz)/Tz] ×100 for concentrations for which Ti<Tz. This gave a scale of 0–200% growth inhibition where 0–100% is for anti-proliferation and 100–200% is for cell killing. Combination activity (synergism) was calculated using the Loewe dose-additivity model as previously described (28,29). This model of additivity provides a null-reference that is predicted by the expected response if the two agents were the same drug. The 3-dimensional model surface, predicted from the two single-agent response curves, is subtracted from the experimentally-derived 3-dimensional dose effect surface to generate a difference volume. This excess matrix volume can be integrated to generate a synergy score. A synergy score cut-off >5 was used to identify combinations of interest in the initial high-throughput screen.

### Western blot assay

Cells were grown in 60-mm dishes and treated with AZD5363, AZD8931 or a combination of the two agents, for the indicated concentrations and times. Cells were washed once with 1 ml ice-cold PBS and then lysed for 15 min on ice in buffer consisting of 25 mmol/l Tris-HCl, 3 mmol/l EDTA, 3 mmol/l EGTA, 50 mmol/l NaF, 2 mmol/l sodium orthovanadate, 0.27 mol/l sucrose, 10 mmol/l B-glycerophosphate, 5 mmol/l pyrophosphate and 0.5% TX-100 and supplemented with protease and phosphatase inhibitors (Sigma). Protein lysates were cleared by microcentrifugation at 13,000 rpm for 10 min at 4°C and the supernatants aliquoted and stored at −80°C. Protein concentration was determined using the Pierce BCA Protein Assay kit (Thermo Scientific). Equivalent amounts of protein (12 μg) from each sample were resolved by SDS-PAGE and transferred by electroblotting onto nitrocellulose membranes (Invitrogen, Carlsbad, CA, USA).

Membranes were then blocked and immunoblotted with the following antibodies overnight at 4°C: HER3, p-HER3 (Y1197), pHER3 (Y1289), HER2, p-HER2 (Tyr1221/1222), AKT, p-AKT (Ser473), p-S6 (Ser235/236), 4EBP1, p-4EBP1 (Ser65), p-FOXO1/3A (Thr24/32), ERK, p-ERK (Thr202/Tyr204), p-NDRG1 (T346), PARP, Cleaved caspase 3, (all from Cell Signaling Technology), Vinculin (Sigma) and p-PRAS40 (Thr246; Invitrogen). After washing three times in PBS-Tween the membranes were then incubated for 1 h at room temperature with anti-mouse or anti-rabbit horseradish peroxidase (HRP)-conjugated secondary antibodies (1:2000; Cell Signaling Technology). Antibody-protein complexes were visualised by chemiluminescence using SuperSignal West Dura Chemiluminescent Substrate (Thermo Scientific) and images captured and quantified using the ChemiGenius system (Syngene, Cambridge, UK)

### Xenograft studies

Mice were bred, maintained and treated in accordance with Institutional guidelines, as previously described (7). HCC1954 cells were harvested from T225 cm tissue culture flasks and a single cell suspension of >90% viability was injected into the left flank of female nude mice (nu/nu:Alpk) in a volume of ~0.1 ml in 50% matrigel. When mean tumour sizes reached ~0.3 cm^3^, the mice were randomized into control and treatment groups. Compounds were dosed by oral gavage in a suspension formulation of hydroxypropylmethylcellulose/Tween-80. The control group received vehicle alone, twice daily by oral gavage. Tumour volumes (measured by caliper), animal body weight and tumour condition were recorded twice weekly for the duration of the study. The tumour volume was calculated (taking length to be the longest diameter across the tumour and width to be the corresponding perpendicular diameter) using the formula: (length × width) × √(length × width) × (π/6). Growth inhibition from the start of treatment was assessed by comparison of the differences in tumour volume between control and treated groups. Since the variance in mean tumour volume data increases proportionally with volume (and is therefore disproportionate between groups), data were log-transformed to remove any size dependency before statistical evaluation. Statistical significance was evaluated using a one-tailed, two-sample t-test. At the end of the study mice were sacrificed by CO_2_ euthanasia. The tumours were harvested and snap-frozen in liquid nitrogen for analysis of protein expression by ELISA.

### ELISA

Frozen tumours were homogenized using FastPrep methodology with FastPrep lysis matrix A (MP Biomedicals, Santa Anna, CA, USA). Lysates were generated in buffer consisting of 25 mmol/l Tris-HCl, 3 mmol/l EDTA, 3 mmol/l EGTA, 50 mmol/l NaF, 2 mmol/l sodium orthovanadate, 0.27 mol/l sucrose, 10 mmol/l B-glycerophosphate, 5 mmol/l pyrophosphate and 0.5% TX-100 and supplemented with protease and phosphatase inhibitors (Sigma). Total and phospho-EGFR, ErbB2 (HER2) and ErbB3 (HER3) were measured by solid phase sandwich ELISA according to the manufacturer’s protocol (R&D Systems, Abingdon, UK).

## Results

### Combining AZD5363 with the EGFR/HER2/HER3 signalling inhibitor AZD8931, results in synergistic growth inhibition in HER2-amplified breast cancer lines

A panel of 23 breast cancer cell lines, representing different segments of breast cancer, was screened to identify cell lines in which AZD8931 synergizes with AZD5363 to inhibit proliferation. The combination was evaluated in a 6×6 dose matrix format, which allows the drug combination activity to be analysed over a wide concentration range. Using a quantitative synergy score, based on the Loewe model of additivity (28), we demonstrate that combination activity is more frequently observed in the HER2-amplified cell lines (Table I). By defining a relative synergy cut-off (synergy score, >5) we found that 6/7 HER2-amplified lines tested show a synergistic interaction between AZD5363 and AZD8931. Interestingly, two triple negative breast cancer lines, MDAMB468 and BT20, which are reported to have amplified EGFR (30) also showed a synergistic interaction.

**Figure 1:**
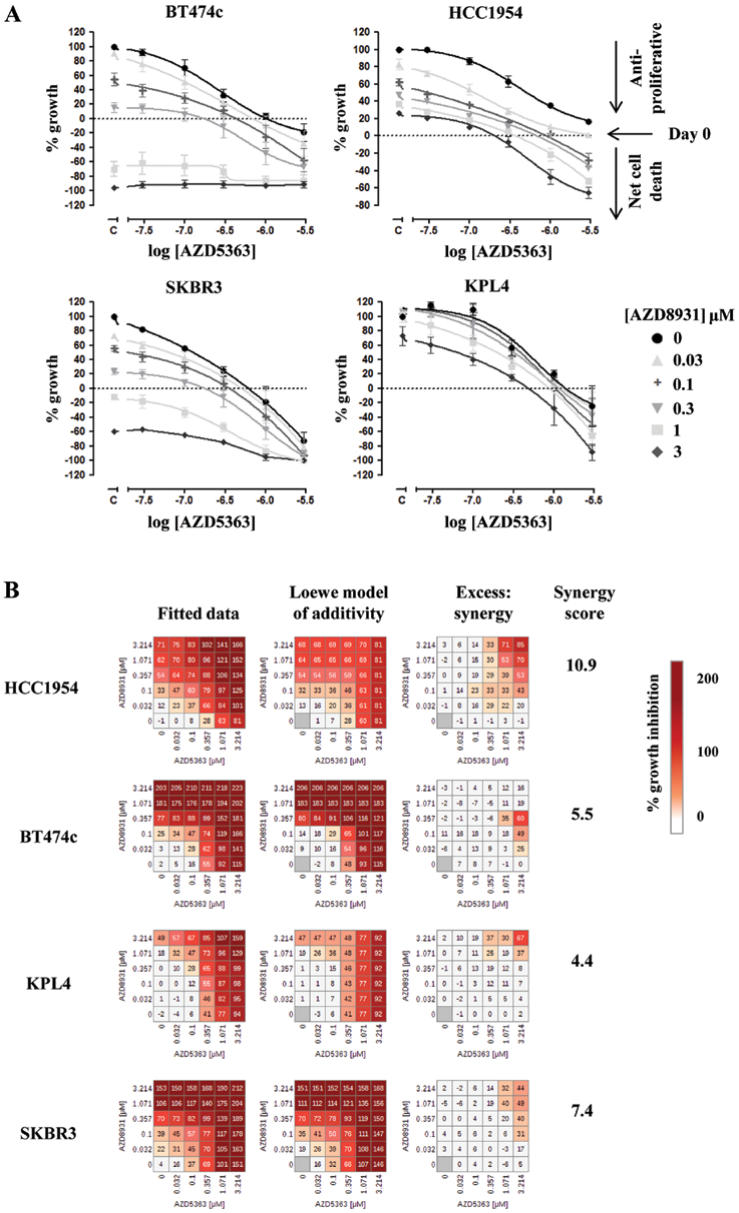
AZD8931 synergises with AZD5363 to inhibit cell growth in HER2-amplified breast cancer cell lines. A panel of HER2-amplified breast cancer cell lines was exposed to increasing concentrations of AZD5363 and AZD8931 in a 6×6 dose response matrix. Live cell number was assessed after 5 days using a sytox green endpoint and synergy scores were generated using the Loewe model of additivity. (A) Growth curves for the combination. Data are expressed as mean % growth ± SEM from 3 independent experiments, where zero growth represents the initial seeding density on the day of dosing. (B) Representative dose matrices and synergy scores from one experiment. Left panel: dose matrix representing percent growth inhibition values taken from the fitted dose response curves. Central panel: Loewe model of additivity calculated from the monotherapy dose response curves. Right panel: Excess heatmap (synergy) calculated by subtracting the Loewe model of additivity data from the fitted data. Calculated synergy scores for each cell line are shown in the far right column.

To confirm the results of the high-throughput screen and further evaluate the effect of combining AZD5363 and AZD8931 we selected a mini-panel of four HER2-amplified breast cancer cell lines and assessed synergistic growth inhibition using the 5-day sytox green assay to measure live cell number. Analysis of the growth curves demonstrated that AZD5363 exerted monotherapy anti-proliferative activity in all of the HER2-amplified lines, as previously described (7) but only induced cell death at concentrations ≤1 μM in two of these lines, BT474c and SKBR3. However, addition of AZD8931 was able to increase the anti-proliferative effects of AZD5363 and cause enhanced cell death over either agent alone in all four of the cell lines tested (Fig. 1A). Analysis of synergism, as described in Materials and methods, suggested that the greatest synergy was observed in HCC1954 cells for this combination (Fig. 1B), with synergy scores of 10.9, 11.4 and 7.8 being generated across the three separate experiments. Mean synergy scores for BT474c, KPL4 and SKBR3 were 4.0, 4.8 and 8.3, respectively.

### AKT inhibition by AZD5363 leads to induction of phospho and total HER2/3 levels

**Figure 2:**
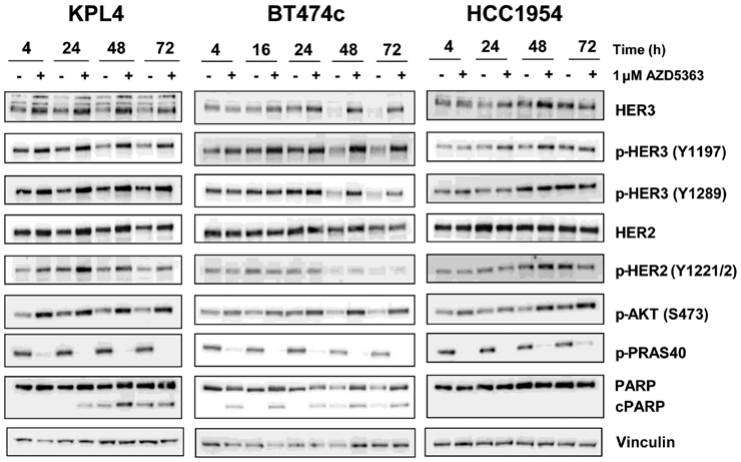
AZD5363 treatment results in feedback upregulation of phospho and total HER2/HER3. Cells were treated with 1 μm AZD5363 for 4–72 h and protein lysates were analysed by immunoblot with the indicated antibodies.

Previous studies have demonstrated that inhibition of the PI3K/AKT/mTOR pathway leads to the activation of compensatory pathways through feedback induction of receptor tyrosine kinases such as HER2 and HER3. To determine whether AZD5363 resulted in similar feedback induction we analysed phospho and total HER2 and HER3 levels, in three of the HER2-amplified lines that had shown good combination effects, following exposure to 1 μM AZD5363 for up to 72 h. This concentration is clinically relevant, as a steady state C_min_ of ~1 μM is achieved throughout the dosing period at the clinically tolerated dosing schedule of 480 mg bid (31). As expected, we observed reproducible, time-dependent increases in phospho and total levels of HER3, and to a lesser extent HER2, in all three of the cell lines examined (Fig. 2).

In addition, pAKT levels were seen to increase. This was not unexpected as it has been reported that binding of an inhibitor to the ATP site of AKT can result in hyperphosphorylation of the kinase in the absence of any pathway feedback effects (32). In contrast, the levels of phospho-PRAS40, a direct substrate of AKT, were markedly decreased, confirming inhibition of AKT activity by AZD5363 at 1 μM. It was unclear why the levels of HER3 appeared to decrease in DMSO-treated BT474c cells over time, but was perhaps due to the cells approaching confluence at the later time points. However, all subsequent experiments assessing total and phosphorylated endpoints were performed at 24 h, when HER3 levels remained constant. Analysis of cleaved PARP levels suggested that whilst 1 μM AZD5363 could induce apoptosis in BT474c and KPL4 cells it was unable to induce cell death as a monotherapy in the HCC1954 cell line. This is consistent with data from the growth curve analysis; in BT474c and KPL4 cultures the cell number fell below the initial seeding density at concentrations of approximately 1 μM, suggesting potent anti-proliferative effects and some cell death, whereas in HCC1954 cultures, 20% growth was still observed even at concentrations as high as 3 μM (Fig. 1A).

### AZD8931 limits RTK feedback induced by AZD5363 and results in more prominent shutdown of downstream signalling pathways and induction of apoptosis

**Figure 3:**
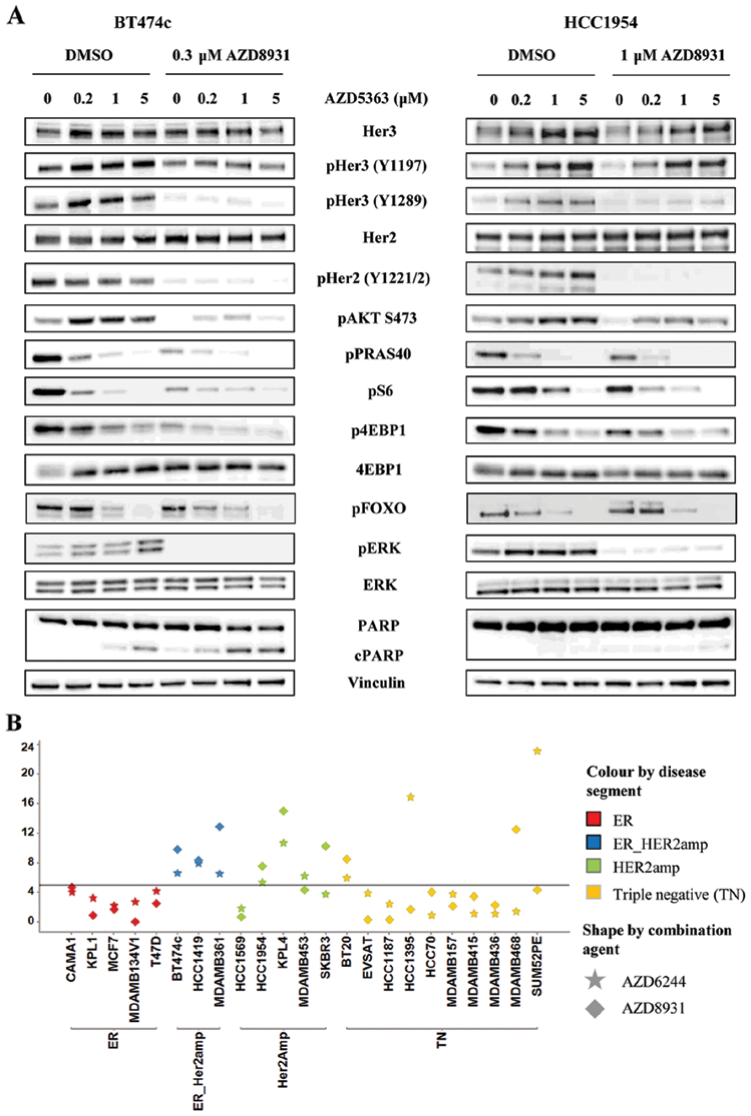
AZD8931 limits AZD5363-induced phosphorylation of HER2/HER3 and results in more prominent shutdown of the PI3K pathway. (A) BT474c or HCC1954 cells were treated for 24 h with increasing concentrations of AZD5363 ±0.3 μM or 1 μM AZD8931, respectively. Protein lysates were analysed by immunoblot with the indicated antibodies. Blots are representative of blots from 2–3 separate experiments. (B) Comparison of synergy scores for the combination of AZD5363 with either AZD8931 or AZD6244.

To determine whether AZD8931 could limit the RTK feedback induced by AZD5363, we assessed the phosphorylation status of HER2 and HER3 as well as several downstream signalling molecules by western blotting in HCC1954 and BT474c cells. In HCC1954 cells, AZD5363 caused a dose-dependent increase in HER2/HER3 phosphorylation at 24 h which was prevented by the addition of 1 μM AZD8931. Phosphorylation of HER2 (Y1221/2) and HER3 (Y1289) was essentially undetectable by western blotting in the presence of AZD8931 (Fig. 3A).

A similar result was obtained in BT474c cells with 0.3 μM AZD8931. This lower concentration was used due to the potency of AZD8931 at inducing cell death in this cell line. Likewise, AZD8931 limited the increase in phospho-AKT which occurs as a result of treatment with AZD5363, returning phospho-AKT back to control levels in both cell lines. This subsequently resulted in more pronounced inhibition of the PI3K/AKT/mTOR signalling pathway as evidenced by a further reduction in PRAS40, S6 and 4EBP1 phosphorylation over either agent alone. PI3K inhibition has also been shown to result in compensatory activation of the ERK signalling pathway, probably at least in part as a consequence of HER2 activation (24). We therefore decided to assess whether AZD5363 increased ERK phosphorylation in BT474c and HCC1954 cells and if so, could this be prevented by the addition of AZD8931. Twenty-four hour exposure to AZD5363 increased phosphorylated ERK in both cell lines. This induction was completely abrogated by the combined treatment with AZD8931. To further investigate whether the induction of phospho-ERK might in part be responsible for limiting the anti-proliferative activity of AZD5363 in HER2-amplified cell lines we tested whether the addition of AZD6244, a MEK1/2 inhibitor, was also able to synergise with AZD5363. Good synergy was observed in several breast cell lines, and this tended to correlate with cell lines which had shown good synergy with AZD8931 (Fig. 3B).

The data in Fig. 1 suggested that combining AZD5363 with AZD8931 caused greater cell death than either agent alone. To determine whether this was due to enhanced apoptosis we analysed cleaved PARP levels by western blotting and demonstrated a greater induction of cleaved PARP in the combination treatment (Fig. 3A). This was particularly evident in the BT474c cells, occurring across the entire concentration range of AZD5363, whereas in HCC1954 cells, induction of cleaved PARP was only really observed at the top concentration of 5 μM.

### Anti-tumour activity of AZD5363 and AZD8931 in the HCC1954 xenograft model

**Figure 4:**
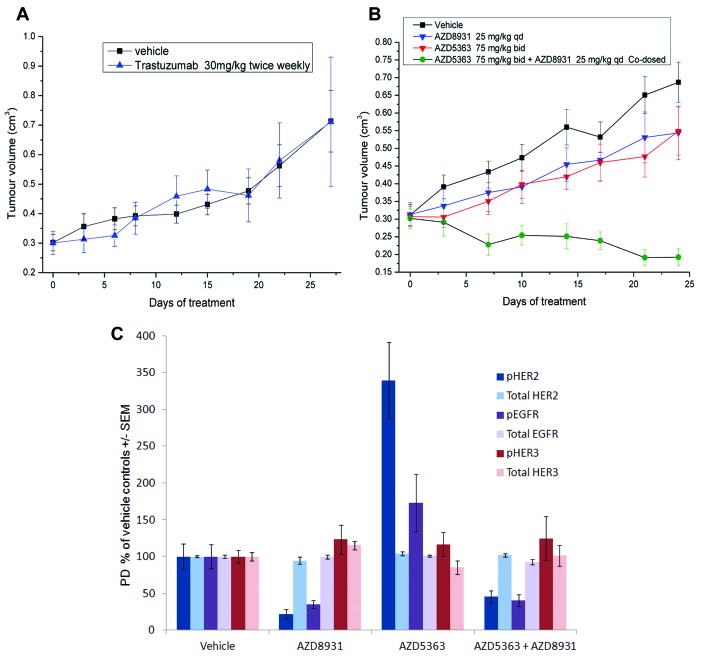
AZD8931 enhances the antitumour effects of AZD5363 in the HCC1954 xenograft model. (A) Mice bearing HCC1954 xenografts were dosed twice weekly with Trastuzumab as indicated. Mean tumour volumes are represented ± SEM. (B) Mice bearing HCC1954 xenografts were randomised to the indicated treatment groups. Mean tumour volumes are represented ± SEM. (C) HCC1954 tumours, collected at the end of study, were lysed and analysed by ELISA with the indicated endpoints. Results are expressed as % vehicle control ± SEM.

To determine whether the enhanced anti-proliferative activity translated *in vivo* we investigated the effects of the combination in the HCC1954 xenograft model, a HER2-amplified, PIK3CA mutant cell line which is resistant to Trastuzumab therapy (Fig. 4A). Monotherapy AZD5363 (75 mg/kg bid) and AZD8931 (25 mg/kg qd) inhibited tumour growth by 42% (p=0.05) and 39% (p=0.07) respectively, compared with vehicle controls (Fig. 4B). In contrast, the combination of AZD5363 and AZD8931 was well tolerated and caused pronounced tumour regression which was sustained for the duration of the dosing period (130% inhibition compared with vehicle controls; p<0.0001 compared with controls and monotherapy groups). To evaluate whether AZD5363-induced feedback to HER receptors was also observed *in vivo* we analysed phospho and total EGFR, HER2 and HER3 levels at the end of anti-tumour study. As expected, AZD8931 inhibited the phosphorylation of HER2 and EGFR, whereas these were increased by AZD5363 monotherapy treatment. The induction of pHER2 and pEGFR by AZD5363 was ameliorated when dosed in combination with AZD8931 (Fig. 4C). There was no significant effect of either agent on phospho HER3 levels *in vivo*.

## Discussion

Numerous PI3K/AKT/mTOR pathway inhibitors, including AZD5363, have progressed into the clinic and are currently being tested in phase I/II clinical trials. To date, modest clinical activity has been observed for these agents when dosed as a monotherapy. Furthermore, data emerging from both preclinical and clinical studies with other targeted therapies suggests that resistance is likely to occur following prolonged treatment. Therefore, a pressing need exists to develop rational combination hypotheses to enhance the therapeutic response and prevent or delay the development of resistance. Relief of feedback to receptor tyrosine kinases has been increasingly demonstrated to be a potential resistance mechanism for inhibitors of the PI3K signalling pathway and in breast cancer this frequently occurs through increased expression and phosphorylation of the HER family of receptors, particularly HER2 and HER3 (16,24–26). Likewise, resistance to trastuzumab (Herceptin) has been associated with increased PI3K signalling, either through hyper-activating mutations in PIK3CA or loss of the tumour suppressor PTEN (19–22). Combined inhibition of PI3K/AKT/mTOR signalling with HER2/HER3 signalling inhibitors is therefore an attractive therapeutic strategy for the treatment of breast cancer.

In this study, we demonstrated that the activity of AZD5363 could be enhanced by the addition of AZD8931, as shown by synergistic growth inhibition *in vitro* and enhanced anti-tumour activity *in vivo*. Using a panel of breast cell lines, representing different segments of disease, we showed that this combination activity was mainly observed in cell lines containing amplified HER2 or in the case of two triple negative lines, amplified EGFR. This is consistent with AZD8931 being a pan EGFR/HER2/HER3 signalling inhibitor.

AKT inhibition resulted in feedback upregulation and phosphorylation of HER3 and to a lesser extent HER2, *in vitro*. This is consistent with previous studies where upregulation of cell surface receptors, including HER3, have been observed in response to a range of PI3K/AKT/mTOR inhibitors (16,18,25). In those studies the activation of FOXO transcription factors, as a direct result of AKT inhibition, was shown to be responsible for the increased expression of RTKs. Given that AZD5363 potently inhibits FOXO phosphorylation and induces translocation of FOXO3a to the nucleus in BT474c cells (Fig. 3) and (7) it is likely that this is the mechanism of HER3 induction in these cells. Indeed, Fox *et al* demonstrated that knockdown of FOXO3a abrogated AZD5363-mediated induction of IGF-1R, IGF-I and IGF-II mRNA in their ER^+^ models of long-term estrogen deprivation (18). Surprisingly, whilst AZD5363 induced the phosphorylation of HER2 and EGFR *in vivo*, the levels of phosphorylated HER3 remained similar to control treated samples. This may suggest that different feedback mechanisms are activated *in vivo*, with receptor activation being heavily dependent on the microenvironment and source of appropriate ligand.

In all cases, addition of AZD8931 prevented the induction of phospho HER2/3 and led to a more prominent suppression of the downstream markers of PI3K pathway signalling. As well as reactivating the inhibited pathway, loss of negative feedback has also been shown to activate parallel signalling networks. For example, inhibition of PI3K/AKT/mTOR with BEZ235 resulted in compensatory activation of ERK signalling which was shown to occur via activation of HER family receptors (24). We observed a similar effect following inhibition of AKT with AZD5363. Twenty-four hour exposure to AZD5363 resulted in a marked increase of phospho ERK in BT474c and HCC1954 cells and this induction was completely prevented by the addition of AZD8931. Furthermore, the MEK1/2 inhibitor, AZD6244, was also able to synergise with AZD5363 in a range of HER2-amplified cell lines, suggesting that activation of ERK signalling in response to AZD5363 treatment may serve as a potential mechanism to limit its efficacy in HER2-amplified breast cancer lines.

Trastuzumab has had a major impact on the treatment of HER2-amplified metastatic breast cancer. However, not all HER2-amplified patients respond to treatment and patients that initially respond eventually develop resistance. Numerous mechanisms of resistance have been identified including hyper-activation of the PI3K signalling pathway, expression of p95HER2 receptor, a truncated form of HER2 which lacks the extracellular ligand-binding domain and dimerisation with other RTKs (33). Several clinical trials, combining PI3K/mTOR inhibitors with HER2 agents in trastuzumab refactory patients have been initiated (www.clinicaltrial.gov; NCT00736970, NCT01471847, NCT00317720, NCT01283789). We have previously demonstrated that AZD5363 enhances the efficacy of both trastuzumab and lapatinib in the KPL4 xenograft model which is only partially sensitive to the HER2 agents as monotherapy (7). Herein, we further show that the combination of AZD5363 and AZD8931 was highly synergistic in HCC1954 cells, a model which expresses high levels of p95HER2 and is resistant to trastuzumab therapy. It is therefore suggested that the combination of AZD5363 with an inhibitor of HER2/HER3 signalling, such as AZD8931, is worth pursuing as a possible treatment option for patients with amplified HER2 who have previously progressed on HER2-directed therapy. The potential application of AZD5363 in the neoadjuvant setting, where patients fail to show a pathologic complete response (pCR) on HER2-directed therapy alone, is also worthy of further evaluation.

## Figures and Tables

**Table I:** Synergy scores for the combination of AZD5363 plus AZD8931 across a panel of breast cancer cell lines representing the different segments of breast cancer.

Cell line	Synergy score	HER2	ER	PIK3CA mutation
CAMA1	4.7	−	+	WT
KPL1	0.9	−	+	E545K
MCF7	1.7	−	+	E545K
MDAMB134V1	0.0	−	+	WT
T47D	2.5	−	+	H1047R
BT474c	**9.8**	+	+	K111N
HCC1419	**8.3**	+	+	WT
MDAMB361	**12.9**	+	+	E545K
HCC1569	0.7	+	−	WT
HCC1954	**7.5**	+	−	H1047R
KPL4	**15.0**	+	−	H1047R
SKBR3	**10.2**	+	−	WT
MDAMB453	4.3	−	−	H1047R
BT20	**8.5**	−	−	H1047R
EVSAT	0.3	−	−	WT
HCC1187	0.3	−	−	WT
HCC1395	1.7	−	−	WT
HCC70	4.0	−	−	WT
MDAMB157	2.1	−	−	WT
MDAMB415	3.4	−	−	WT
MDAMB436	2.3	−	−	WT
MDAMB468	**12.5**	−	−	WT
SUM52PE	4.3	−	−	WT

Cells were treated with increasing concentrations of AZD5363, AZD8931 or the combination of both in a 6×6 dosing matrix and cell number was measured after 5 days of treatment using a sytox green assay. Synergy scores were generated using the Loewe model of additivity. A synergy score of >5 (bold) was achieved predominantly in cells expressing amplified HER2.
